# Cloud droplet activation of organic–salt mixtures predicted from two model treatments of the droplet surface†
†Electronic supplementary information (ESI) available. See DOI: 10.1039/c8em00345a


**DOI:** 10.1039/c8em00345a

**Published:** 2018-10-10

**Authors:** Jack J. Lin, Jussi Malila, Nønne L. Prisle

**Affiliations:** a Nano and Molecular Systems Research Unit , FI-90014 University of Oulu , P. O. Box 3000 , Oulu , Finland . Email: nonne.prisle@oulu.fi

## Abstract

The droplet surface plays important roles in the interaction between organic aerosols with clouds and climate. Surface active organic compounds can partition to the droplet surface, depleting the solute from the droplet bulk or depressing the droplet surface tension. This may in turn affect the shape of the droplet growth curve, threshold of aerosol activation into cloud droplets, activated droplet size distributions, and cloud radiative effects. In this work, a new monolayer model along with a traditional Gibbs adsorption isotherm model was used in conjunction with equilibrium Köhler theory to predict cloud condensation nuclei (CCN) activation of both simple and complex surface active model aerosol systems. For the surface active aerosol considered, the monolayer droplet model produces similar results to the Gibbs model as well as comparable results to CCN measurements from the literature, even for systems where specific molecular identities and aqueous properties are unknown. The monolayer model is self-contained and fully prognostic, and provides a versatile, conceptually simple, yet physically based model for understanding the role of organic surfactants in cloud droplet formation.

## Abstract 2

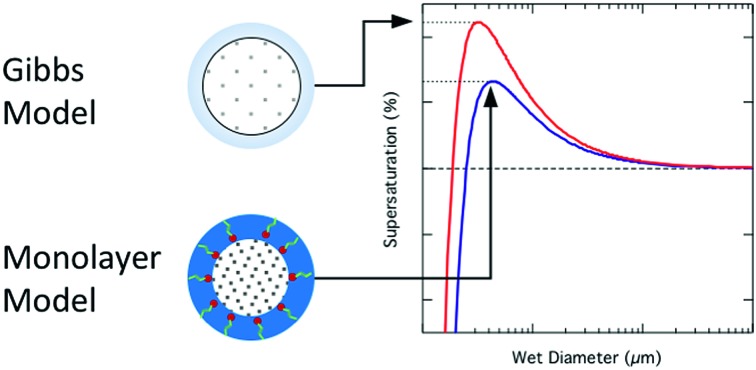
A new monolayer model predicts the bulk-surface partitioning, surface composition, and thickness of droplets comprising chemically unresolved, atmospherically relevant organic aerosols.

##

Environmental significance

Many atmospheric organic compounds are surface active. The partitioning of these compounds in droplets is an important process that can have impacts on surface tension depression and critical supersaturation. Understanding the role of the processes that occur at droplet surfaces can lead to the reduction in the uncertainty in the anthropogenic indirect effect. We compare the performance of a monolayer surface model to a traditional model based on Gibbs thermo-dynamics. The new monolayer model requires fewer compound-specific inputs making it more suitable for studying chemically unresolved atmospheric 5 organic aerosols. It adds to our understanding of the processes that control surface activity in cloud droplets and presents a new pathway to constrain surface impacts on cloud activation and radiative balance.

## 1 Introduction

The role of surfactants in cloud droplet activation has been a recurring theme for modelling of organic aerosol cloud climate interactions.^1^–^12^ While it has been established that surface activity can impact single droplet activation through both lowering surface tension and from diminishing the solute effect by surface partitioning,^3^–^6^,^12^,^13^ the relative impact of these effects and their potential synergies under different conditions are currently not well constrained.^9^,^10^,^14^,^15^ This balance has been shown to significantly impact the predictions of global cloud droplet numbers and radiative forcing^16^ and is therefore important to understand in a fully prescriptive way. Disregarding surfactant effects has in several cases been shown to give correct predictions of measured droplet critical supersaturation, similar to comprehensive model frameworks.^4^,^5^,^13^ However, surfactant effects change the shape of the droplet growth curve^3^–^6^ and changes in the activated droplet size spectrum and large-scale radiative effects should therefore also be accounted for.

Droplet models based on Gibbs adsorption thermodynamics^3^,^5^ and their approximations^6^,^17^,^18^ have been relatively successful in predicting cloud activation for a few selected model surfactant systems but require knowledge of molecular identity and properties, such as molecular weight and pure bulk phase mass density, for all droplet components and composition-dependent properties, such as surface tension and water activity, for the full range of droplet solution states considered. Therefore, previous studies have largely been limited to a few well-characterized surfactant systems (see *e.g.* Petters and Petters^8^ and references therein). In particular, industrial surfactants with relatively well-characterized molecular and solution properties, such as sodium dodecyl sulfate (SDS), Triton, and Zonyl, have been the subject of a number of studies as model systems for atmospheric surfactants. SDS in particular has been a favored surface-active organic aerosol model compound, both by itself and in binary aerosol mixtures with NaCl, in experimental and theoretical studies of cloud condensation nuclei (CCN) activation.^1^,^3^,^5^,^8^,^19^,^20^ Of atmospherically relevant surfactants, previous studies have focused on straight-chain fatty acids and their carboxylate salts^4^,^5^,^11^ and less surface active dicarboxylic acids.^21^–^24^ Recently, more complex surface active mixtures, including atmospheric limonene-derived organosulphates,^25^ organic mixtures coating pollen grains called pollenkitt,^12^ and proxies for atmospheric humic-like substances (HULIS),^13^ have been interpreted in a Gibbs model framework to varying degrees of success.

Several alternative droplet frameworks to the Gibbs approach for CCN activation thermodynamics have been proposed.^6^,^9^,^10^,^26^,^27^ The common feature of these frameworks is the representation of the droplet surface as a physical (mono-)layer instead of a mathematical dividing surface with related excess quantities as done in the Gibbs models. Recently, Malila and Prisle^27^ developed a relatively simple physical monolayer droplet model for predicting bulk/surface partitioning of all species in the droplet, yielding specifically the composition and thickness of the surface phase. The model predictions can therefore be directly compared not only to experimentally observed CCN activity, but also to experimental and computational studies of the droplet structure. The monolayer model is completely self-contained and involves no tunable parameters in addition to thermodynamic input data. It also requires fewer inputs of specific thermodynamic data than the Gibbs droplet models. In particular it does not require explicit knowledge of droplet water and solute activities, which are often not known or even well-defined for atmospherically relevant organic mixtures.

Here, we use the monolayer droplet model of Malila and Prisle^27^ in combination with Köhler^28^ theory to predict the CCN activity of organic aerosol systems with different surface activity, surfactant strength and chemical complexity. We compare model predictions to the Gibbs models of Prisle *et al.*^5^ and Prisle and Molgaard when available, to experimental data from the literature.

## 2 Methods

We consider CCN activation under two different thermodynamic frameworks used to evaluate the bulk/surface partitioning equilibrium of surface active and other droplet components. In the monolayer droplet model, partitioning occurs into a physical surface layer with thickness *δ* defined by its volume-weighted components. In the Gibbs model, partitioning is evaluated with respect to a conceptual two-dimensional dividing surface between uniform droplet liquid and gas phases and the deviation of droplet thermodynamics from that of a system comprised of these two bulk phases is described in terms of surface excess quantities. A detailed comparison of these approaches is given in ref. 27. With either choice of droplet framework, critical conditions for droplet activation are then evaluated using Köhler theory.^28^

### 2.1 Monolayer model

Bulk and surface compositions are related to the surface tension according to the Laaksonen–Kulmala^30^,^31^ equation,1
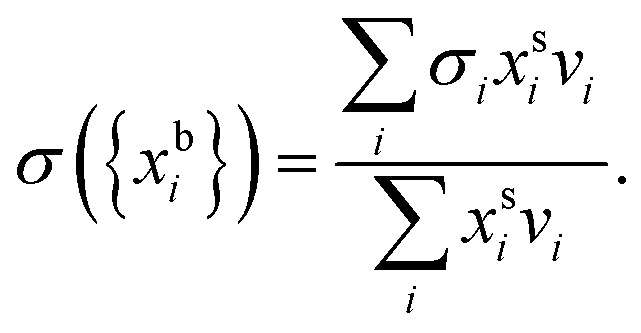
Here, 
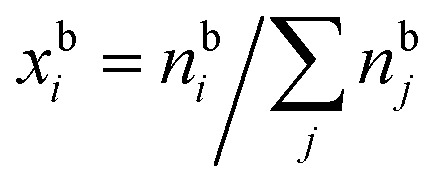
 and 
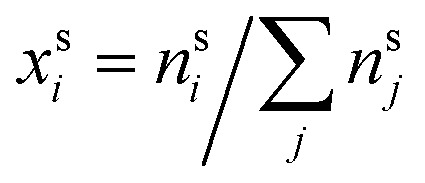
 are the bulk and surface mole fractions, corresponding to molar amounts *n*b*i* and *n*s*i*, of droplet component *i*, and *σ* (without a subscript) refers to the surface tension of the solution phase, while *σ*_*i*_ and *v*_*i*_ are the surface tensions and liquid-phase molecular volumes of each (pure) component. Expressing the surface phase thickness2
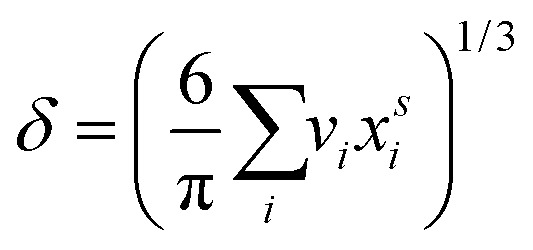
and droplet radius *R* as functions of {*n*b*i*} and {*n*s*i*} by imposing the condition of conservation of mass, partitioning of each component in the droplet between the bulk and surface is evaluated iteratively.^27^ While eqn (1) and (2) can be derived by assuming an ideal mixture of molecules with different sizes, they are used here as semi-empirical relations with molecular volumes from densities and surface tensions obtained from measurements in the literature thereby capturing real solution behaviour. The systems were selected based on the availability of experimental ternary surface tension data. In a few cases where no corresponding experimental ternary solution density data were available, ideal pseudo-binary mixing of an organic surfactant with salty water was assumed for obtaining composition-dependent *v*_*i*_.

### 2.2 Gibbs model

In the Gibbs model, surface excesses or adsorptions *Γ*_*i*_ (the surface excess number area density of molecules) of droplet components are related *via* Gibbs' adsorption equation. Following earlier work,^3^–^5^ we write this equation as3
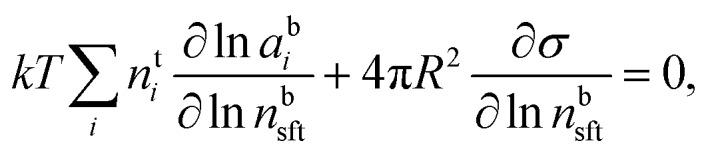
where *n*bsft is the number of surfactant (“sft”) molecules in the droplet bulk, *n*t*i* the total amount of component *i* in the droplet (bulk and surface) phase, and *a*b*i* the bulk activity of *i*. Eqn (3) is solved numerically by assuming volume additivity (such that the droplet radius *R* is given by the sum of individual pure component molar volumes 
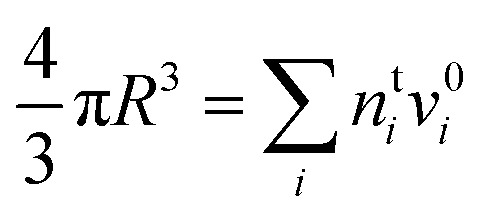
) and conservation of mass (*n*s*i* + *n*b*i* = *n*t*i*) to evaluate the bulk and surface excess mole numbers of different species, *n*b*i* and 4π*R*^2^*Γ*_*i*_, respectively. Furthermore, a constraint is imposed that the ratio of water and salt molecules in the bulk and surface remains fixed, to reduce the number of independent variables. The assumption of volume additivity, *i.e.* that partial molar volumes *v*_*i*_ are equal to those of pure substances *v*0*i* irrespective of droplet phase composition, is an expression of ideal mixing properties for the droplet solution and a thermodynamic feature treated differently by the monolayer and Gibbs droplet models in this work. However, as liquid-phase densities of ternary systems in the monolayer model are approximated with those of ideal binary mixtures of aqueous salt solution and surfactant, it is not likely to be a significant source of difference between different frameworks. Following the approach documented by Prisle *et al.*,^5^ we also assume mixing ideality in the form of unity activity coefficients in eqn (3) for all components in the droplet bulk (*a*b*i* = *x*b*i*).

A major difference considering surface partitioning in the monolayer and Gibbs models is that the Gibbs dividing surface has no volume, *i.e.*
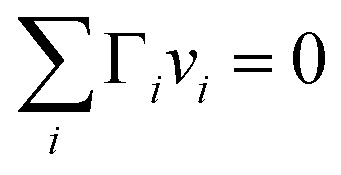
, so that molecules adsorbed on the surface do not contribute to the total volume 
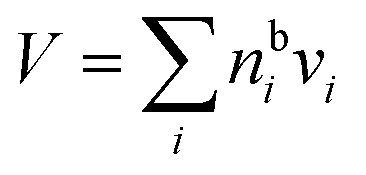
 of the droplet. Moreover, the surface excess quantities are sensitive to the position of the Gibbs dividing surface, which is implicitly defined here from the total bulk molecular amounts solved from eqn (3) to yield the specified total volume of the droplet. Therefore, a positive surfactant surface excess must be balanced by a total negative surface excess of other droplet components, here water and salt. In Section 3.1.3 we discuss the implications of this for comparing the extent of surface partitioning evaluated with the two different surface models. More details can be found in ref. 5.

### 2.3 Köhler theory

Droplet activation is evaluated in terms of the critical saturation ratio (*S*_crit_, often presented as an excess percentage *i.e.* saturation ratio (*S* – 1) × 100%) for water vapour, iterated as the maximum of the Köhler curve for equilibrium growth of a spherical droplet4
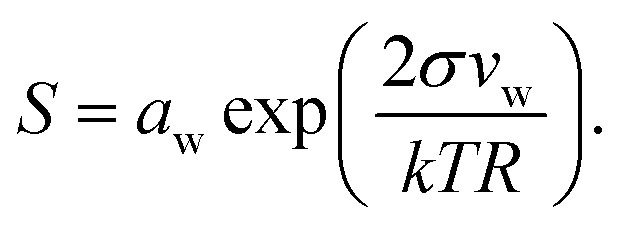



In eqn (4), *a*_w_ = *a*bw is the water activity and *v*_w_ the partial molecular volume of water in the droplet (bulk), and *k* and *T* are the Boltzmann constant and temperature in kelvin, respectively.

For complex mixtures, experimental data for the water activity are often unavailable. Instead, *a*_w_ may then be obtained from computational estimates such as group-contribution methods or, in the case of dilute solutions, approximated with the bulk mole fraction of water, *x*bw. While group contribution methods have been shown to give accurate activity coefficients for binary organic aqueous mixtures of atmospheric interest,^32^,^33^ there are only a few studies on their validity for organic–inorganic aqueous mixtures,^34^,^35^ and in general there is a significant spread between the estimated activity coefficients from different models. For chemically unresolved organic mixtures such as model-HULIS and pollenkitts, it is not possible to use group-contribution methods at all. Here, we therefore in all cases approximate the water activity with the bulk mole fraction of water (*a*bw = *x*bw) for consistency and to facilitate comparison between the different systems. Droplet *x*bw and *σ* are determined from the bulk mole fractions derived from the monolayer and Gibbs models and used in eqn (4) to evaluate the properties of droplets during growth and activation.

### 2.4 Model systems

Four different surface active organic aerosol model systems are considered in this study. Succinic acid (SCA) and SDS represent simple molecular surfactants with different surfactant strength in macroscopic solutions. Nordic Aquatic Fulvic Acid (NAFA) and pollenkitts are complex and chemically unresolved surface active mixtures with different water solubility and surface activity. Cloud droplet activation is calculated for particles consisting of each of these organics mixed with inorganic salts – sodium chloride (NaCl) for SCA, SDS, and NAFA and ammonium sulphate (AS, (NH_4_)_2_SO_4_) for pollenkitts. These salts were selected based on the availability of experimental ternary surface tension data for the surface active organics of interest (see Table 1).

**Table 1:** Properties of the studied aqueous mixtures

Surfactant	Salt	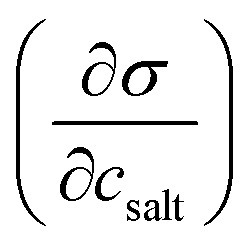 (N m^–1^ [c]^–1^)^*a*^	*a* (N m^–1^)	*b* ([c])	*Δ*_sft_ (nm)
NAFA	NaCl	1.61 × 10^–3^^*b*^	*a*_0_ + *a*_1_*ε* + *a*_2_*ε*^2^^*c*^	*b*_0_ + *b*_1_*ε* + *b*_2_*ε*^2^^*c*^	2.04
Succinic acid	NaCl	See Vanhanen *et al.*,^29^ eqn (4)–(6)	0.63		
Sodium dodecyl sulphate^*d*^	NaCl	1.61 × 10^–3^^*b*^	13.9 × 10^–3^^*e*^	9.273 × 10^–6^/(9.733 × 10^–3^ + *c*_salt_)^*e*^	0.93
Poplar pollenkitt	(NH_4_)_2_SO_4_	16.55 × 10^–6^^*f*^	3.53 × 10^–3^^*f*^	0.18 × 10^–4^^*f*^	1.01
Ragweed pollenkitt	(NH_4_)_2_SO_4_	16.55 × 10^–6^^*f*^	3.37 × 10^–3^^*f*^	0.23 × 10^–4^^*f*^	1.30

^*a*^For NaCl and SDS, [c] = M, while for the all other compounds [c] = kg m^–3^.

^*b*^ Ref. 5.

^*c*^ Ref. 14: here *ε* is the mass fraction of NAFA in the dry particle, *a*_0_ = 72.1344 × 10^–3^, *a*_1_ = –158.4 × 10^–3^, and *a*_2_ = 93.52 × 10^–3^; and *b*_0_ = 6.5559, *b*_1_ = –15.51, and *b*_2_ = 9.431.

^*d*^For SDS–NaCl solution, surface tension at a critical micelle concentration (CMC) of 36 mN m^–1^ was used in calculations.

^*e*^ Ref. 1.

^*f*^ Ref. 12.

Cloud droplet activation of SDS has been studied extensively in previous work, and SDS mixtures are included here for reference, to benchmark calculations with the new monolayer droplet model. NAFA is a commercially available mixture used in previous studies as model HULIS.^13^,^14^,^36^ HULIS is a class of macromolecular compounds so named due to their resemblance to humic and fulvic acids from terrestrial and aquatic sources.^37^ Significant amounts of HULIS have been isolated from ambient particulate matter from a variety of environments,^38^–^41^ and they are known to be surface active and depress the surface tension of aqueous solutions.^42^–^44^ The remaining organics studied here have been identified as constituents of CCN relevant aerosols in the lower atmosphere. SCA is a slightly soluble (58–100 g L^–1^) organic and one of the more abundant dicarboxylic acids observed in the atmosphere.^45^–^51^ Previous studies have demonstrated the ability of whole pollen grains^52^,^53^ and fragments of pollen grains^54^ to act as CCN. The pollen grains of certain plant species are coated with a viscous material called pollenkitt.^55^ The composition of pollenkitt varies across species and is chemically diverse,^56^ but may resemble other atmospheric organic aerosol mixtures in terms of solubility and surface activity in aqueous solution.^12^ In this work, we focus on pollenkitt extracted from the pollen of black poplar (*Populus nigra*) and common ragweed (*Ambrosia artemisiifolia*), which were found to be the most and least CCN and surface active, respectively, among six pollenkitts studied by Prisle *et al.*^12^

### 2.5 Surface tension and treatment of micelles

Relevant input data needed for model calculations for these aerosol mixtures were obtained from the literature and are summarised in Table 1. The densities of all ternary aqueous solutions were calculated as ideal binary mixtures of the organic compound with water–salt solutions.^57^,^58^ Solution surface tensions, with the exception of water–NaCl–succinic acid mixtures,^29^ were evaluated from an augmented Szyszkowski–Langmuir relation,5
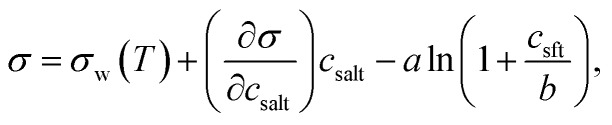
where *c*_salt_ is the bulk concentration of NaCl or (NH_4_)_2_SO_4_, *c*_sft_ is the bulk concentration of surfactant, and *a* and *b* mixture-specific adsorption parameters given in Table 1. Also given are the monolayer thicknesses for pure surfactants *Δ*_sft_ evaluated from eqn (2). For a more detailed description of the thermodynamic input data for the calculations, see ref. 12, 13 and 27.

Any composition dependent surface tension relation relying on measured surface tension isotherms, including eqn (5), breaks down above the critical micelle concentration (CMC) where the surface tension–concentration relation changes discontinuously. A detailed, thermodynamically consistent treatment of micelle formation in aqueous droplets and impact on cloud activation is highly non-trivial. We therefore adopt a simplified, phenomenological approach by applying the condition *σ* = *σ*_CMC,sft_ both for droplets reaching surface saturation by forming a full monolayer in the monolayer model and for bulk concentrations reaching the CMC in either droplet framework. This allows us to extend the calculations to droplets at bulk concentrations above the ternary surfactant CMCs. With a perfect description of droplet thermodynamics, these states should coincide, but since the monolayer concept is a simplification, this will likely not be fully realized in the current framework. The simplification has the further advantage of circumventing the need for knowing pure surfactant surface tensions for input to the monolayer model. For example, pure SDS is an amorphous solid at room temperature.

## 3 Results & discussion

In the following sections, we present modelled results for droplet critical supersaturation *S*_crit_, surface tension *σ*_crit_ and growth factor at activation GF_crit_ = *d*_crit_/*d*_dry_, organic partitioning *n*ssft/*n*tsft (monolayer model) or 4π*R*^2^*Γ*_sft_/*n*tsft (Gibbs model), and surface phase thickness *δ* (only the monolayer model) as functions of dry particle size *d*_dry_ and composition in terms of dry particle surfactant mass fraction *ε*_sft_. For all systems, calculations are made for dry particles with diameters 50–150 nm and covering the full range of dry particle surfactant mass fractions *ε*_sft_ ∈ [0,1]. For clarity, only results for selected particle compositions and sizes are shown in the following sections. When available, experimental CCN activity data from the literature are shown for further comparison.

### 3.1 Succinic acid

#### 3.1.1 Critical supersaturation

**Fig. 1:**
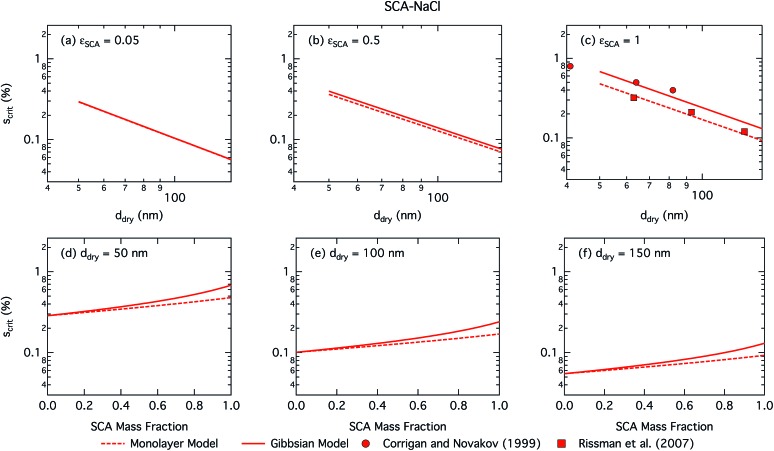
Critical supersaturations calculated with the monolayer and Gibbs droplet models as a function of dry particle size for SCA mass fractions (a) 0.05; (b) 0.5; (c) 1 and as a function of SCA mass fraction for dry particle sizes (d) 50 nm; (e) 100 nm; and (f) 150 nm. Measured critical supersaturations as a function of pure SCA dry size from Corrigan and Novakov^59^ and Rissman *et al.*^60^ are also shown in panel (c).

Fig. 1 shows critical supersaturations for SCA–NaCl particles as a function of dry particle size and SCA mass fraction. In each case, results are shown for *d*_dry_ = 50, 100, and 150 nm and *ε*_SCA_ = 0.05, 0.5, and 1.

Succinic acid particles are fairly CCN active, with predicted *S*_crit_ in the range 0.055–0.48% for the monolayer model and 0.055–0.68% for the Gibbs model. The monolayer model predicts somewhat smaller *S*_crit_—or greater CCN activity—than the Gibbs model, with the difference between the models growing for increasing *ε*_SCA_ and *d*_dry_. A general feature in this work is how the monolayer model predicts larger bulk concentrations of surfactant at a given droplet size and composition, due to the constraint on the extent of surface partitioning imposed by the finite thickness of the physical surface layer and finite densities of pure and aqueous solutes.^27^ This effect becomes more pronounced for larger SCA fractions, where the monolayer is more fully saturated, and for larger particle sizes and activating droplets, where smaller surface/bulk volume ratios mean that the partitioning mass balance is not as strongly shifted toward the surface phase, as for smaller particles and droplets with larger surface/bulk volume ratios. These effects are discussed in further detail in Section 3.1.2. Nevertheless, the overall *S*_crit_ values are similar for the two models and converge as expected in the binary limit of a pure NaCl dry particle without surface active SCA. This occurs even though in the monolayer model NaCl is partially excluded from the surface layer, causing a slight increase in the bulk concentration of NaCl for a given droplet size. However, this difference is seen to have a negligible effect on the modelled droplet properties for SCA–NaCl particles.

The CCN activity of succinic acid mixed with other dicarboxylic acids and NaCl has been determined experimentally,^61^–^63^ together with several studies^22^,^59^,^60^,^64^–^67^ reporting *S*_crit_ values for pure succinic acid particles. We have shown experimental data for pure succinic acid as a function of dry succinic acid particle size from Corrigan and Novakov^59^ and Rissman *et al.*^60^ in Fig. 1. Both experiments generated succinic acid particles from water solution, drying the aqueous droplets, and size selecting the dried particles before measuring their CCN activity. Curiously, data of Corrigan and Novakov^59^ agree well with the results from the Gibbs model while data of Rissman *et al.*^60^ agree well with the results from the monolayer model. This illustrates how experiment–model closure is affected by not only the choice of model framework, but also potentially unresolved experimental conditions.

#### 3.1.2 Surface tension of activating droplets

**Fig. 2:**
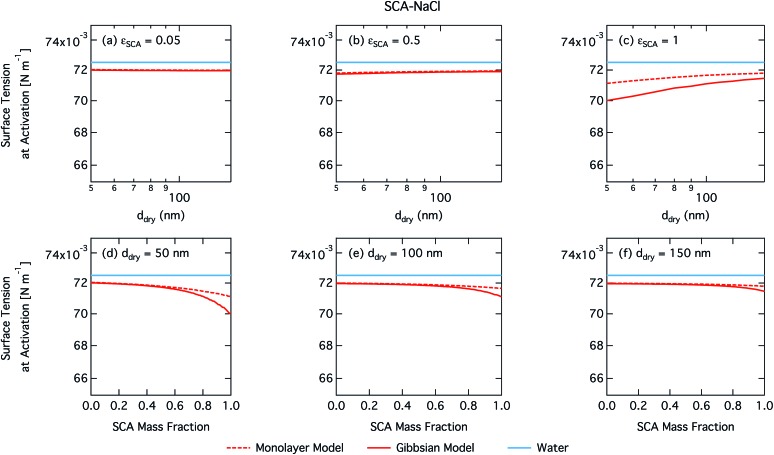
Surface tension at activation calculated with the monolayer and Gibbs droplet models as a function of dry particle size for SCA mass fractions (a) 0.05; (b) 0.5; (c) 1 and as a function of SCA mass fraction for dry particle sizes (d) 50 nm; (e) 100 nm; and (f) 150 nm.

**Fig. 3:**
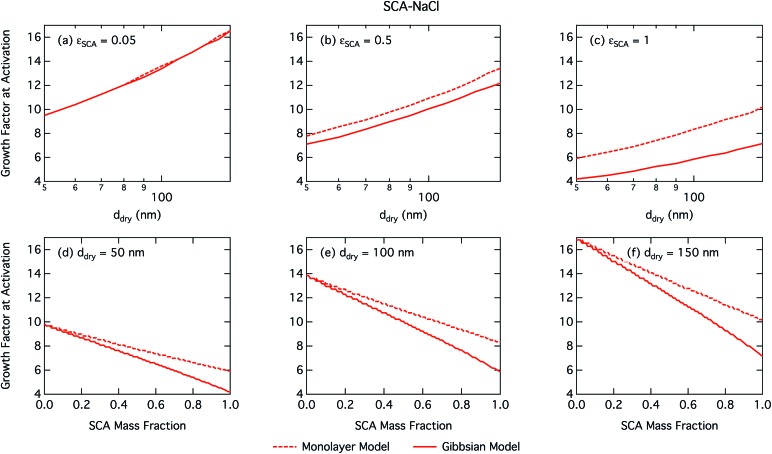
Droplet growth factor at activation calculated with the monolayer and Gibbs models as a function of dry particle size for SCA mass fractions (a) 0.05; (b) 0.5; (c) 1 and as a function of SCA mass fraction for dry particle sizes (d) 50 nm; (e) 100 nm; and (f) 150 nm.

Calculated droplet surface tension at the critical point is shown in Fig. 2 as a function of dry particle size and SCA mass fraction. Despite SCA having weak to moderate surfactant strength (ability to lower surface tension) in macroscopic aqueous solution,^68^ the surface tensions of activating droplets are not significantly reduced from that of pure water, except for dry particles with the smallest sizes and highest fractions of SCA. For these particles, droplets form the most concentrated solutions when they are activated. This is reflected in Fig. 3, which shows the diameter growth factor at droplet activation GF_crit_, again as a function of dry particle size and SCA mass fraction. The growth factor at droplet activation increases monotonically with dry particle size for a given composition, and with salt fraction (decreasing *ε*_SCA_) for a given particle size, meaning the droplets are larger and more dilute at activation. It has previously been noted that as the dry particle size increases for a given initial composition, droplets are more and more dilute at the point of activation,^69^ also in the case of particles comprising surfactants.^5^,^6^,^13^

Activation growth factors for other particle mixtures follow similar trends as seen for SCA and are given in the ESI†.

#### 3.1.3 Surface partitioning

**Fig. 4:**
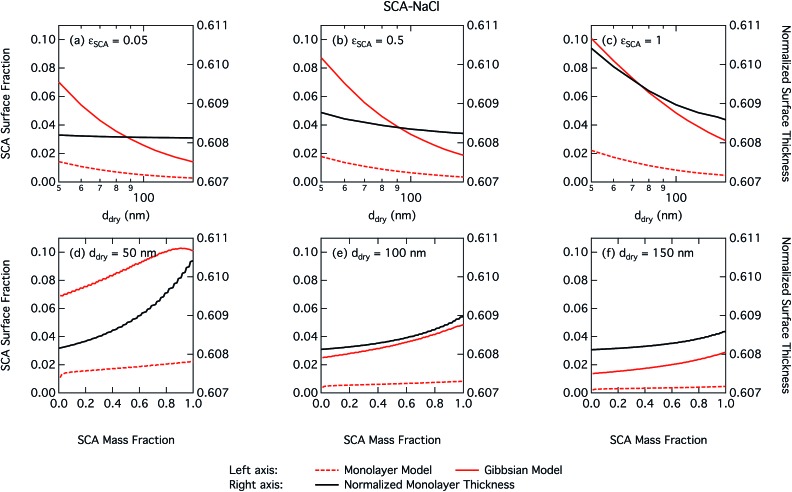
Droplet SCA surface fraction on the left axes calculated with the monolayer and Gibbs models and surface thickness from the monolayer model normalized to the thickness of one SCA monolayer on the right axes, as a function of dry particle size for SCA mass fractions (a) 0.05; (b) 0.5; (c) 1 and as a function of SCA mass fraction for dry particle sizes (d) 50 nm; (e) 100 nm; and (f) 150 nm.

Differences between the two droplet models in predicted surface tension and, as a consequence, predicted critical supersaturation stem from the evaluated position of the partitioning equilibrium in the droplets. Fig. 4 shows the fraction of total SCA molecules *n*tSCA in the droplet phase that reside in the surface at the critical point of droplet activation as a function of dry particle size and SCA mass fraction. These numbers conceptually represent somewhat different quantities for the two droplet models. In the monolayer model, the surface concentration represents the total number of molecules in the monolayer with finite thickness and the SCA surface fraction is given as *n*sSCA/*n*tSCA, whereas in the Gibbs model the partitioning is given in terms of the surface excess of molecules with respect to the defined Gibbs dividing surface and the surface fraction is evaluated from the surface excess as 4π*R*^2^*Γ*_SCA_/*n*tSCA. In both cases, the surface fraction is sensitive to the assumed properties of the surface. For the monolayer model, the finite surface layer thickness and liquid-phase density of surface components impose a physical limitation or restriction on the extent of surface partitioning. In the Gibbs framework, the surface excess quantities of molecules do not contribute to the droplet volume and therefore no similar physical volume constraint is imposed on the partitioning mass balance. However, the surface excess amounts of molecules are implicitly sensitive to the defined position of the surface, which constrains the mass balance of partitioning to yield the liquid phase bulk volume equal to the specified droplet volume. For both surface frameworks, the conservation of mass means *n*tSCA = *n*sSCA + *n*bSCA or *n*tSCA = 4π*R*^2^*Γ*_SCA_ + *n*bSCA for the respective bulk mole numbers of SCA.

As already mentioned, due to the physical limitation on the number of molecules that can partition into the surface monolayer imposed by the finite volume and component densities, this droplet model consistently predicts lower SCA surface fractions (and therefore higher SCA bulk concentrations) compared to the Gibbs model. Also shown on the right axes of Fig. 4 are the surface thicknesses *δ* calculated from the monolayer model, normalized to the estimated thickness of one full SCA monolayer *Δ*_SCA_ (Table 1). Unsurprisingly, the droplet monolayer thickness increases with increasing surface fraction, typically with increasing SCA mass fraction in the dry particle and decreasing dry particle size. This effect is smaller when droplets are more dilute, as seen from the growth factors in Fig. 3.

It is tempting to compare the thickness of the surface monolayer to that of a pure substance phase corresponding to the evaluated surface excess of surfactant in the Gibbs framework. For example, Petters and Petters^32^ predict surface excesses corresponding to several monolayers for surfactants stronger than those considered here. However, a positive surfactant surface excess evaluated in the Gibbs model must be balanced by a total negative excess of these other solution components to yield the condition 
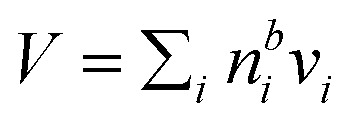
. Assigning the surfactant surface excess of the Gibbs model to a volume based on the properties of a pure surfactant phase is therefore somewhat ambiguous in terms of attributing the volume from components with negative surface excess (water and salt), and accommodating only the surface excess of surfactant into a surface layer would result in a non-equilibrium droplet bulk composition. Here, we therefore illustrate the difference between the two surface model frameworks in terms of evaluated partitioning of surfactant, specifically between the surface and bulk.

### 3.2 SDS

SDS is a much stronger surfactant in macroscopic solutions than succinic acid and predicted trends for most droplet properties are very similar but more pronounced for SDS particles compared to SCA.

#### 3.2.1 Critical supersaturation

**Fig. 5:**
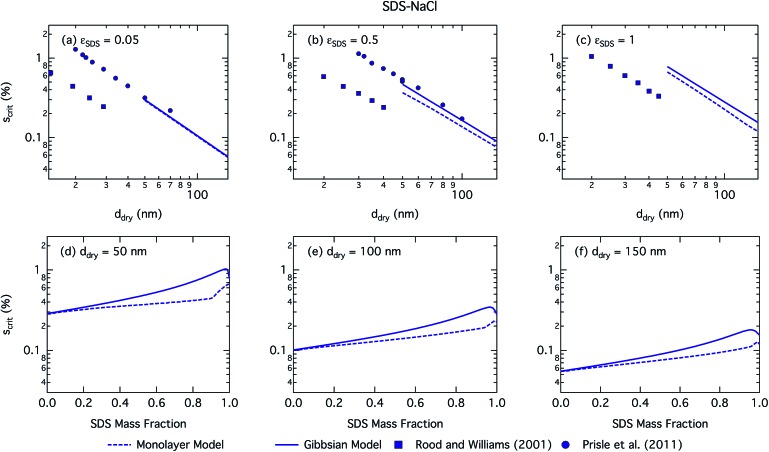
Critical supersaturations calculated with the monolayer and Gibbs models as a function of dry particle size for SDS mass fractions (a) 0.05; (b) 0.5; (c) 1 and as a function of SDS mass fraction for dry particle sizes (d) 50 nm; (e) 100 nm; and (f) 150 nm. Measured critical supersaturations as a function of dry particle size from Rood and Williams^19^ and Prisle *et al.*^6^ are also shown in panels (a)–(c). The measurements in panel (a) are for pure NaCl and are shown for comparison.

Fig. 5 shows the critical supersaturations for mixed SDS–NaCl particles as a function of dry particle size and SDS mass fraction. The absolute differences between values predicted with the two droplet models are larger than for succinic acid. Again, predicted *S*_crit_ is consistently higher with the Gibbs model compared to the monolayer model and increases with *ε*_SDS_ except for the very highest SDS fractions. This is again due to the differences resulting from restricted *vs.* unrestricted surface partitioning of SDS in the two models, leading to higher bulk concentrations of SDS in the monolayer model, and thus lower surface tension in droplets at any given size and overall composition. For activating droplets, this effect is further modulated by the dilution state, as given by the droplet growth factor (Fig. S1 in the ESI†), and the resulting water activity from both SDS and NaCl, as well as the effect of a larger droplet surface area or volume, for the Gibbs and monolayer models, respectively, to bulk volume ratio on enhancing partitioning to the surface phase.

**Fig. 6:**
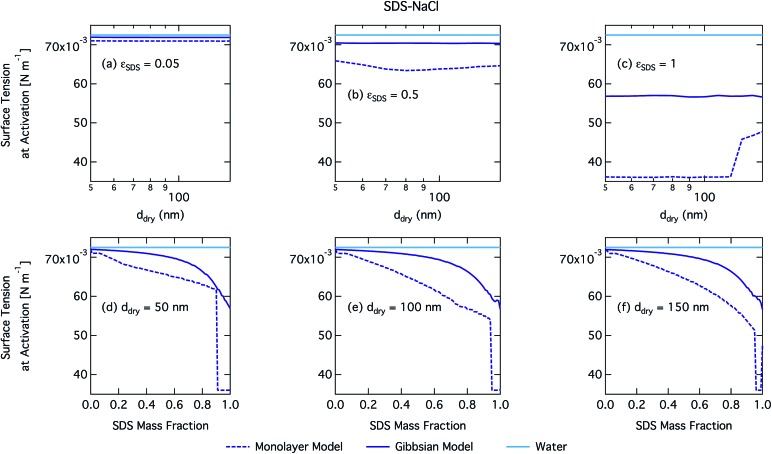
Surface tension at activation calculated with the monolayer and Gibbs models as a function of dry particle size for SDS mass fractions (a) 0.05; (b) 0.5; (c) 1 and as a function of SDS mass fraction for dry particle sizes (d) 50 nm; (e) 100 nm; and (f) 150 nm.

We notice a bump in *S*_crit_*vs. ε*_SDS_ for SDS mass fractions around 0.95–1.00 as previously predicted using the Gibbs model^5^,^6^ due to salting out of SDS by a small amount of NaCl in the high surfactant fraction (and relatively higher overall concentration, see Fig. S1 in the ESI†) range. This effect is much less prominent for the monolayer model predictions, where the CMC is reached in droplet bulk for SDS mass fractions 0.92 (for a 50 nm droplet) and 0.97 (for a 150 nm droplet), as seen from the predicted droplet surface tension in Fig. 6 and further discussed below. The salting-out effect of NaCl on SDS could be overestimated in the Gibbs model, due to a computationally exaggerated so-called common ion effect resulting from the ideal water activity assumption. It is possible that this effect may be entirely a computational feature. For example, Prisle *et al.*^70^ and Öhrwall *et al.*^71^ both found no indication of an actual common ion effect in surface sensitive XPS studies of aqueous surfactant-salt solutions. In other words, no enhanced salting out of ionic surfactants with Na^+^ counterions was seen in Na^+^ salt mixtures compared to other salts of similar ionic strength.

The CCN activity of SDS mixed with NaCl has been experimentally determined by several groups.^5^,^8^,^19^,^72^ Experimental data from Rood and Williams^19^ and Prisle *et al.*^6^ are also shown in Fig. 5. The experimental data in panel (a) of Fig. 5 are for pure NaCl particles, but are included in the panel for illustrative purposes. The data from Prisle *et al.*^6^ agree well with the Gibbs model consistent with previous studies.^3^ The data of Rood and Williams^19^ show much lower critical supersaturations than predicted by either model.

#### 3.2.2 Surface tension of activating droplets

**Fig. 7:**
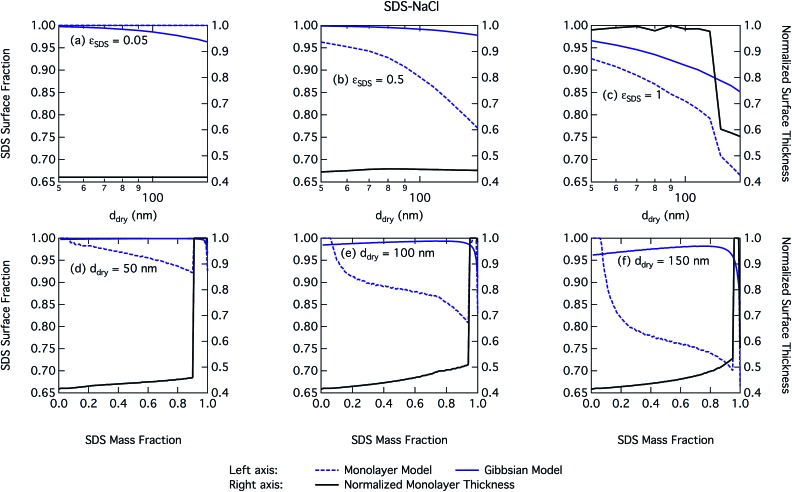
Droplet SDS surface fraction on the left axes calculated with the monolayer and Gibbs models and surface thickness from the monolayer model normalized to the thickness of one SDS monolayer on the right axes as a function of dry particle size for SDS mass fractions (a) 0.05; (b) 0.5; (c) 1 and as a function of SDS mass fraction for dry particle sizes (d) 50 nm; (e) 100 nm; and (f) 150 nm.

Fig. 6 shows droplet surface tension at activation as a function of SDS–NaCl dry particle size and SDS mass fraction. The dependency on both the particle size and mixing state is similar but much stronger for SDS than for SCA, due to the greater surface activity of SDS. The surface tension of activating droplets is significantly decreased at high SDS mass fractions and for large dry particle sizes. For the smallest particles, enhanced surface partitioning (Fig. 7) due to the large droplet surfaces has a greater influence on droplet bulk concentration than the significantly higher total droplet concentration predicted in terms of smaller critical growth factors (Fig. S1†).

Again, the monolayer model predicts lower droplet surface tensions than the Gibbs model, due to higher bulk concentrations in droplets of a given size and overall composition from restricted surface partitioning. With the monolayer model, the noted sharp drop in activation surface tension predicted for droplets with SDS mass fractions of 0.92–0.97 represents droplet bulk compositions reaching the CMC for SDS. This is not observed for the Gibbs model, where predicted bulk concentrations are much lower due to more pronounced depletion from unrestricted surface partitioning (see Fig. 7). The reason for the discontinuous drop in surface tension at activation to its value at the CMC is the simplified condition *σ* = *σ*_CMC,SDS_ applied in the monolayer model both for droplets reaching surface saturation and when the bulk concentration reaches the CMC, as mentioned in Section 2.4.1. The sharp drop in surface tension is more pronounced for smaller droplets. Because the surface in the monolayer model has a finite thickness, the surface/bulk volume ratio tends to infinity as the droplet radius decreases towards the surfactant monolayer thickness. For a sufficiently large surface/bulk volume ratio, partitioning essentially becomes a step function in the sense that for systems with CMC or solubility limitations, a single molecule in the bulk simultaneously reaches the maximum thermodynamically possible concentration and causes the maximum deviation of surface tension from that of pure water.^27^

#### 3.2.3 Surface partitioning

The fractions of the total SDS solute present in the droplet surfaces at activation is shown in Fig. 7. With both droplet models, the majority of SDS is partitioned to the surface for all particle systems studied. As the SDS mass fraction for a given dry particle size increases, in the monolayer model it eventually reaches the CMC as described above. The mass fraction where this occurs decreases slightly with decreasing dry particle size reflecting the influence of increasing droplet concentration. For pure SDS particles in panel (c) of Fig. 7, the activated droplets become dilute enough that the surface layer in the monolayer model is no longer saturated. This can be seen from the step increase in the growth factor in Fig. S1 in the ESI† and also produces a step increase in the surface tension at activation for the droplet. This behaviour is a consequence of the lower hygroscopicity of pure SDS particles when compared to mixed SDS–NaCl particles.^3^,^8^

### 3.3 Complex surfactants

The monolayer model has a significant advantage compared to Gibbs models in general that fewer compound- and composition-specific parameters for the mixtures are needed and therefore must be known or assumed.^27^ Specifically, no activity coefficients, which are very hard to obtain for most aqueous mixtures of atmospheric organic aerosols, are needed for the evaluation of bulk/surface partitioning within the droplet. All information on intermolecular interactions in the droplet phase is taken implicitly into account using composition dependent density and surface tension functions. These properties are challenging, but still often feasible, to measure with significant accuracy. This is particularly relevant for increasingly complex and atmospherically relevant systems, as for sparse ambient aerosol samples, where sufficient amounts of material for resolving concentration dependent aqueous properties are usually not obtained. Here, we study droplet properties during growth and activation for two surface active complex organic aerosol systems, NAFA, which does not have a well-defined molecular structure,^73^–^75^ and pollenkitts, which are each diverse mixtures of different organic compounds with unknown specific molecular identity and mixing ratios.^12^,^56^,^76^–^78^ These organic aerosol mixtures have previously been studied within the Gibbs droplet model framework^12^,^13^ and the necessary thermodynamic data are therefore available to make model predictions feasible with both droplet models used here.

#### 3.3.1 NAFA

**Fig. 8:**
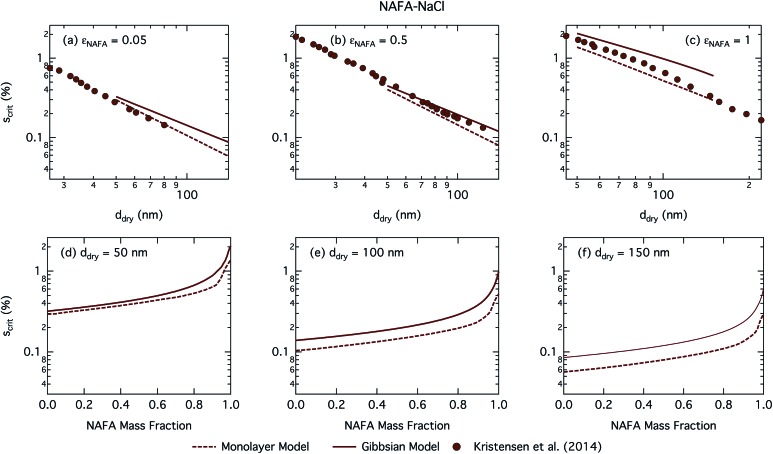
Critical supersaturations calculated with the monolayer and Gibbs models as a function of dry particle size for NAFA mass fractions (a) 0.05; (b) 0.5; (c) 1 and as a function of NAFA mass fraction for dry particle sizes (d) 50 nm; (e) 100 nm; and (f) 150 nm. Measured critical supersaturations as a function of dry particle size from Kristensen *et al.*^14^ are also shown in panels (a)–(c). The measurements in panel (a) are for pure NaCl and are shown for comparison.

Fig. 8 shows the critical supersaturation as a function of dry particle size and NAFA mass fraction. Predicted trends in *S*_crit_ are similar to those of SCA and SDS, although with a less significant salting out effect than for SDS in the monolayer model and none in the Gibbs model. No bump in the critical supersaturation is observed in contrast to SDS, indicating no additional salting out effect on droplet activation. The predicted trends in activation behavior are very similar for the two droplet models. As before, the monolayer model predicts more CCN active particles than the Gibbs model. Absolute differences between the models grow for increasing dry particle sizes, with a slight effect of increasing NAFA mass fraction for the smaller particles.

Fig. 8 shows experimental data from Kristensen *et al.*^14^ In general, their measured critical supersaturations fall somewhere in between the predictions of the two models. Smaller dry particles are activated at smaller critical droplet sizes than larger particles with the same overall composition (see Fig. S4† for growth factors.) The smaller droplets have a larger surface area to bulk volume ratio for the Gibbs model or surface to bulk volume ratio for the monolayer model than larger droplets. The mass balance of surfactant partitioning between the droplet bulk and surface is sensitive to the surface/bulk ratio for given molecular and solution properties, leading to a stronger enhancement of surface partitioning in the smaller droplets. The measured data are closer to those of the Gibbs model at smaller dry particle sizes where this effect is more pronounced and closer to that in the monolayer model for larger dry particles where the effect is less pronounced.

**Fig. 9:**
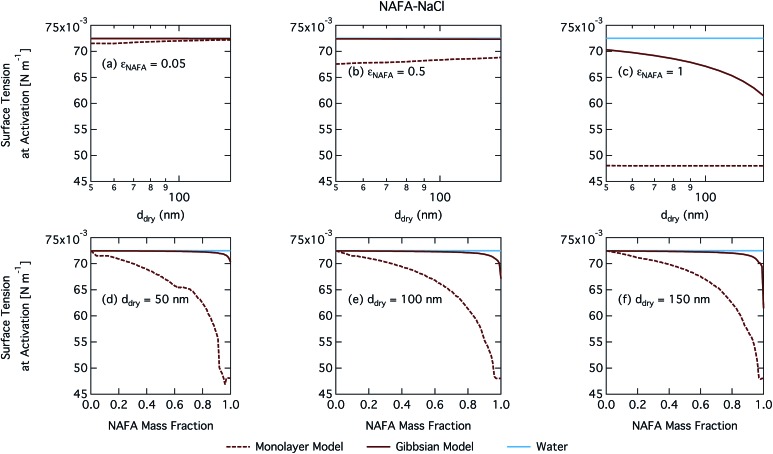
Surface tension at activation calculated with the monolayer and Gibbs models as a function of dry particle size for NAFA mass fractions (a) 0.05; (b) 0.5; (c) 1 and as a function of NAFA mass fraction for dry particle sizes (d) 50 nm; (e) 100 nm; and (f) 150 nm.

**Fig. 10:**
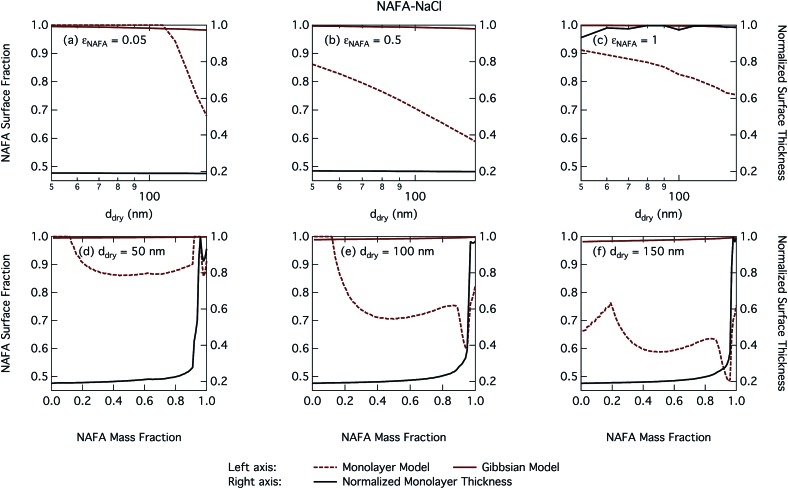
Droplet NAFA surface fraction on the left axes calculated with the monolayer and Gibbs models and surface thickness from the monolayer model normalized to the thickness of one NAFA monolayer on the right axes as a function of dry particle size for SCA mass fractions (a) 0.05; (b) 0.5; (c) 1 and as a function of NAFA mass fraction for dry particle sizes (d) 50 nm; (e) 100 nm; and (f) 150 nm.

The CCN activity of NAFA has previously been modelled taking surface tension, non-ideality, and surface partitioning into account with the Gibbs model.^13^ The Gibbs model was run here with a different ternary surface tension parameterisation (equation form and fitting parameters) than used by Prisle and Molgaard^13^ for consistency between the model conditions within this work. Fig. 9 shows the droplet surface tension at activation as a function of dry particle size and NAFA mass fraction. The predicted surface tensions show large differences between the two models. While the Gibbs model predicts very little reduction in droplet surface tension at the point of activation and only for particles with the highest mass fractions of NAFA, surface tensions predicted with the monolayer model are significantly reduced at all *ε*_NAFA_. This is again due to a clear difference in the droplet bulk composition predicted with the two different partitioning schemes, as seen in Fig. 10. Unrestricted surface partitioning in the Gibbs model under most conditions leads to significantly greater depletion of the droplet bulk. Here, essentially all NAFA solute is partitioned to the surface of activating droplets, but at the same time there is almost no surface tension depression, because the droplet bulk is nearly completely depleted. In both frameworks, surface tension and water activity are described as functions of droplet bulk composition. Physically, as the partitioning is established as an equilibrium gradient of surface active solute between surface and bulk phases, complete depletion of the bulk is only approached asymptotically.

From Fig. 9, the surface tension predicted at droplet activation by the monolayer model is seen to increase slightly with dry particle size, but on the other hand decreases significantly with increasing dry particle NAFA mass fraction. The increasing trend with dry size follows from the concurrent overall increasing dilution state of activating droplets (Fig. S4 in the ESI†). For increasing NAFA mass fractions, droplets are overall more concentrated at activation (Fig. S4†). In addition, the surface layer thickness (Fig. 10) increases more rapidly with increasing NAFA mass fraction for smaller dry particles. At some of the highest NAFA mass fractions (*ε*_NAFA_ ≥ 0.95), the relatively large NAFA molecules – with a reported average molar mass of 4266 g mol^–1^ (ref. 79) – are excluded from the surface layer, causing droplet bulk concentrations to approach the NAFA CMC. As the CMC is reached, a full monolayer coverage of NAFA at the surface is predicted from eqn (1), with the surface tension of pure NAFA approximated as *σ*_CMC,NAFA_ = 48 mN m^–1^. The same phenomenon is also observed, although much less pronounced, for SCA (Fig. 2 and 4).

The peculiar non-monotonic variation of NAFA surface fraction with *ε*_NAFA_ for a fixed dry particle size predicted by the monolayer model (Fig. 10(d)–(f)) partly stems from the assumed form of surface tension parameters. As the surface fraction predicted by the monolayer model is derived from measurements of surface tension, the model is sensitive to how these physical observations are parameterised (see the ESI† for the full sensitivity study.) Because droplets are finite and confined systems, the partitioning mass balance is a sensitive function of droplet size, modulating both the dilution state and position of the surface/bulk partitioning equilibrium of the surfactant. This sensitivity in mass balance is further affected by the nonlinear dependence of surface thickness on composition and translates into a sensitive surface tension dependency on these conditions. It is possible that the sensitive non-monotonic variation with dry particle parameters seen in Fig. 10 would also be evident in actual droplet systems, but, to our knowledge, no experiment can currently resolve such a dependence. Interestingly, this behavior is not reflected in an equally dramatic variation in either *S*_crit_, critical radius or surface tension of activating droplets.

#### 3.3.2 Pollenkitt

The predicted CCN activation behavior of the two pollenkitt mixtures is very similar for both droplet models and for brevity, only results for ragweed pollenkitt are therefore shown here. The discussion for poplar pollenkitt follows that of ragweed below, and corresponding figures can be found in the ESI†.

**Fig. 11:**
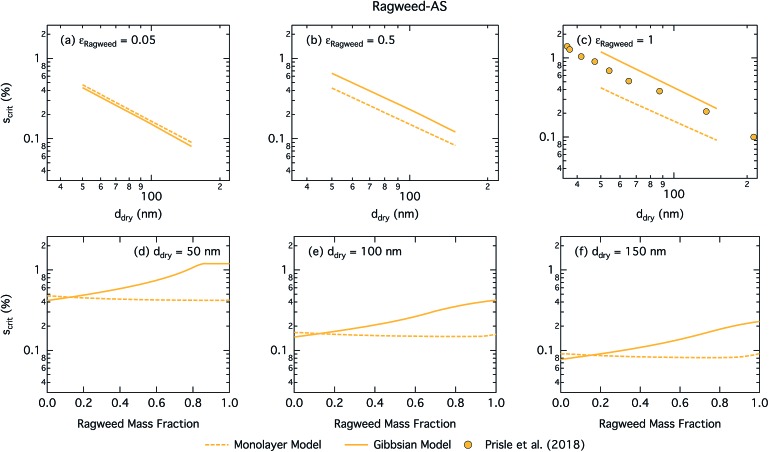
Critical supersaturations calculated by using the monolayer and Gibbs models as a function of dry particle size for ragweed pollenkitt mass fractions (a) 0.05; (b) 0.5; (c) 1 and as a function of ragweed pollenkitt mass fraction for dry particle sizes (d) 50 nm; (e) 100 nm; and (f) 150 nm. Measured critical supersaturations as a function of pure ragweed pollenkitt dry particle size from Prisle *et al.*^12^ are also shown in panel (c).

Fig. 11 shows the critical supersaturation for ragweed pollenkitt–ammonium sulphate particles as a function of dry particle size and ragweed pollenkitt mass fraction. As seen with the other surface active organic aerosol systems, the Gibbs model also here predicts higher critical supersaturations than the monolayer model, except for ragweed mass fractions less than 0.12. The difference between the two models remains fairly constant with dry particle size, but becomes noticeably greater for larger *ε*_ragweed_. There is a crossover between critical supersaturations predicted with the two models for *ε*_ragweed_ between 0.1 and 0.2. This signature is also found in the critical growth factors (Fig. S3 in the ESI†) and may be related to the different treatment of non-surface active, *i.e.* salt, components in the two models as described in Section 2. As differences in critical supersaturation for the two models can be seen even for the binary AS salt case (*ε*_ragweed_ = 0), this may be due to differences in aqueous densities predicted when salt composition differs. In the monolayer model, droplets also have some AS salt in the surface, thereby decreasing the bulk concentration and Raoult effect from the salt. These differences can change the shape of the droplet growth (Köhler) curves and are further modulated by somewhat different droplet sizes at activation (see Fig. S3 in the ESI†). As *ε*_ragweed_ increases, these effects become relatively less significant compared to other effects of pollenkitt partitioning, leading to the observed crossover of predictions from the two models.

Measurements of critical supersaturation as a function of ragweed pollenkitt dry size from Prisle *et al.*^12^ are also shown in panel (c) of Fig. 11. Both predictions from both models and the measurement data have a very similar slope in log *d*_dry_ – log *S*_crit_ space. For pure pollenkitt particles, the Gibbs model predicts critical supersaturations closer to those seen in the measurements.

**Fig. 12:**
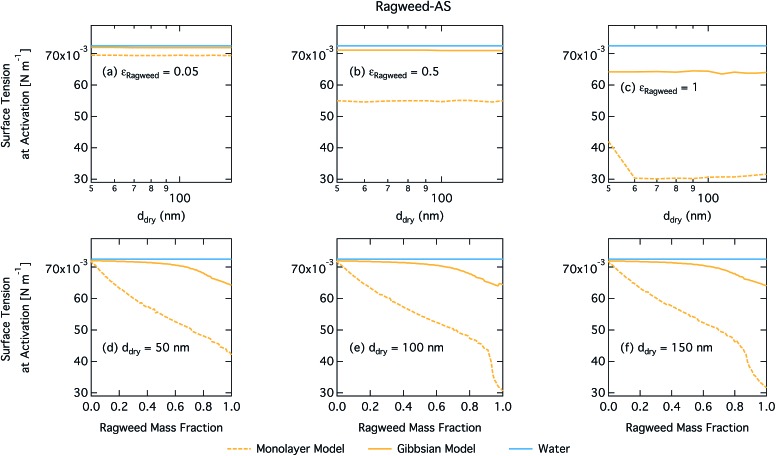
Surface tension at activation calculated by using the monolayer and Gibbs models as a function of dry particle size for ragweed pollenkitt mass fractions (a) 0.05; (b) 0.5; (c) 1 and as a function of ragweed pollenkitt mass fraction for dry particle sizes (d) 50 nm; (e) 100 nm; and (f) 150 nm.

**Fig. 13:**
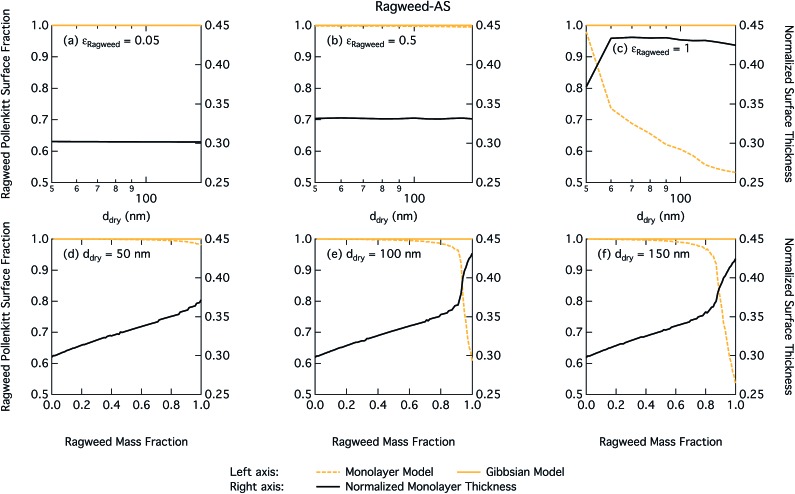
Droplet ragweed pollenkitt surface fraction on the left axes calculated by the monolayer and Gibbs models and surface thickness from the monolayer model normalized to the thickness of one ragweed pollenkitt monolayer on the right axes as a function of dry particle size for pollenkitt mass fractions (a) 0.05; (b) 0.5; (c) 1 and as a function of pollenkitt mass fraction for dry particle sizes (d) 50 nm; (e) 100 nm; and (f) 150 nm.

Droplet surface tensions at the critical point are shown in Fig. 12 as a function of dry particle size and ragweed pollenkitt mass fraction. Surface tensions predicted with the monolayer model are consistently lower than those predicted with the Gibbs model. In the monolayer model, droplet surface tension is significantly reduced at all particle sizes and compositions, even reaching the imposed CMC condition for pure ragweed particles above 60 nm, whereas the reduction predicted with the Gibbs framework is much more modest and only becomes significant for larger dry particle sizes and *ε*_ragweed_. The surface tensions of macroscopic pollenkitt solutions were measured by Prisle *et al.*^12^ for binary aqueous pollenkitt and for pollenkitt–ammonium sulphate mixtures with a pollenkitt mass fraction of 0.8. The ternary pollenkitt–ammonium sulphate aqueous tension parameterisation is therefore unconstrained at lower pollenkitt mass fractions, and similarly for droplets with lower *ε*_ragweed_, even in the absence of surface partitioning. Fig. 13 shows very high degrees of pollenkitt surface partitioning predicted for both droplet frameworks. These values may be exaggerated by bias in the surface tension parameterisation from measurements at high *ε*_ragweed_, where salting out effects may be more significant. Strong changes in salting out are not seen in Fig. 11, but may not be evident when conditions of low *ε*_ragweed_ are not represented in the surface tension parameterisations.

### 3.4 General discussion

**Fig. 14:**
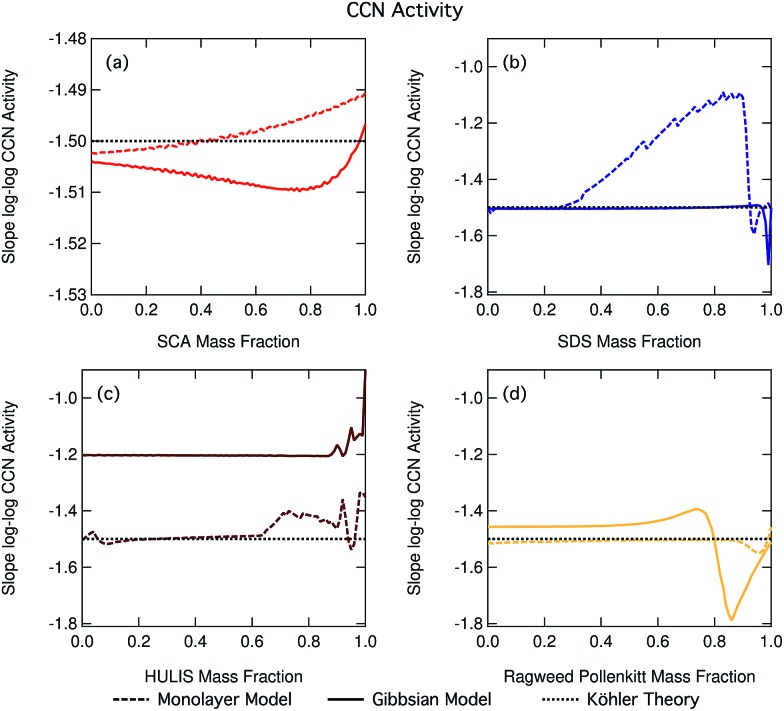
Slopes of log–log plots of critical supersaturation *versus* dry diameter as a function of organic mass fraction in dry particles, for (a) SCA, (b) SDS, (c) NAFA, and (d) ragweed pollenkitt. Note the different scale on the *y*-axis for SCA in panel (a).

Fig. 14 shows the slopes of log–log plots of critical supersaturation as a function of dry particle size plotted as a function of surfactant mass fraction for each of the studied aerosol systems. The constant log *d*_dry_ – log *S*_crit_ slope value of –1.5 predicted using basic equilibrium Köhler theory is shown in each panel for reference. Deviations of the slope from this value are indicative of the presence of droplet effects which introduce a different dependency of *S*_crit_ on *d*_dry_ than predicted using basic Köhler theory. We clearly see such deviations for many of our studied particle mixtures and the trend in deviation from the –1.5 line is greatly different between the different organic aerosol systems. In the case of SDS and ragweed pollenkitt particles, major deviations also exist between predictions with the two droplet partitioning models. The small fluctuations seen for some of the traces are numerical noise in the model.

In general, a number of effects can affect droplet growth and activation at the same time with competing size dependencies. In our present calculations, dry particle size and composition are varied in a well-known and systematic fashion, and we have assumed ideal droplet solutions such that activity coefficients do not vary with droplet dilution. Therefore, potential effects changing the log *d*_dry_ – log *S*_crit_ slope are concentration dependent droplet surface tension reduction and water activity increase from bulk/surface partitioning.

As seen in the growth factor variation with dry particle size (Fig. 3 and S3–S6 in the ESI†), larger particles are generally activated as larger, more dilute droplets. From the Köhler eqn (4) it is therefore seen that concentration dependent droplet surface tension and surface partitioning effects on water activity will lead to opposite dependencies of *S*_crit_ on *d*_dry_ in the absence of further modulating effects. Concentration dependent surface tension at the critical droplet size will increase as larger particles are activated into larger, more dilute droplets, leading to increasing *S*_crit_ and a less steep (greater than –1.5) slope of log *d*_dry_ – log *S*_crit_. Surface partitioning will increase *a*_w_ and *S*_crit_ more strongly for the smaller droplets, leading to a steeper (smaller than –1.5) log *d*_dry_ – log *S*_crit_ slope. Therefore, we can overall say that if surface tension effects are dominating the size dependency of *S*_crit_, the slope of log *d*_dry_ – log *S*_crit_ will be less steep than the value of –1.5 predicted using basic equilibrium Köhler theory and if surface partitioning depletion effects on *a*_w_ dominate, the log *d*_dry_ – log *S*_crit_ slope will conversely be steeper. We see that, in general, both situations come into play across the full range of particle compositions studied in this work.

When the slopes in Fig. 14 remain constant at a value of –1.5, the size dependency of *S*_crit_ is the same for all particle compositions as would have been predicted using basic Köhler theory without accounting for effects of surface activity. This, however, does not mean that the absolute values of *S*_crit_ are also the same, only the dependency on dry particle size. It also does not indicate the absence of surfactant effects, only that the overall influences of any size dependent effects present must balance out for all dry particle compositions to yield the same dependency of *S*_crit_ on *d*_dry_ as in the absence of these effects. This is the case for nearly all mass fractions of SDS in the Gibbs model and pollenkitt in the monolayer model. For succinic acid, there are small slope changes for predictions with both models (note the different scale on the axis, compared to the other three panels).

When the log *d*_dry_ – log *S*_crit_ slope deviates from –1.5 but remains constant across dry particle composition for a given set of surfactant–salt mixtures, particles display a different size dependency of *S*_crit_ than predicted using basic Köhler theory, but this size dependency remains unchanged across the surfactant–salt composition range. Thus, among any size dependent effects which are present, the overall balance of these effects in producing the size dependency of *S*_crit_ does not change across the ensemble of surfactant–salt mixtures. Specifically, the balance between surface tension and partitioning depletion at droplet activation changes with dry particle size, changing the log *d*_dry_ – log *S*_crit_ slope value from –1.5, but this size dependent change otherwise remains the same for particles with different compositions. This can be seen for most of the Gibbs model results, for the major part of the NAFA particles, and in some sections of the monolayer model results for SDS and ragweed. The particle composition regions where there is a large size-dependent effect correspond to the transition regions in terms of CCN activity where it therefore becomes especially important to characterize both the surface tension and partitioning in an explicit and decoupled manner.

When there is a change in the slope of log *d*_dry_ – log *S*_crit_ with dry particle composition, the overall balance or relative importance between effects that introduce different size dependencies of *S*_crit_ changes as a function of surfactant mass fraction in the particles. Here, specifically the balance of size dependencies in *S*_crit_ introduced by surface tension and bulk/surface partitioning changes as the dry particle composition changes. Such changes are seen for SDS particles with the monolayer model as SDS fractions increase beyond 0.25, with the Gibbs model for the very highest surfactant mass fractions, and for NAFA and ragweed pollenkitt in both models, especially for larger organic fractions. The modality of log *d*_dry_ – log *S*_crit_ slope change is however very different between the systems and models shown in Fig. 14.

Fig. 14 shows the effects of multiple processes and process levels. The large differences seen in the behavior of different surface active organic aerosol systems reflect the complex nature and impact of surface activity on droplet properties and cloud activation across particle sizes and compositions. These results underscore the importance of developing a more thorough understanding of atmospheric surfactants and their role in determining organic aerosol CCN activity.

#### 3.4.1 Comparison to other partitioning models

The surface monolayer droplet model as an alternative to the Gibbs framework makes predictions that are not too different except in the very sensitive CCN transition regimes identified in Fig. 14. However, implications are significant for cloud microphysics. Facchini *et al.*^2^ calculated that a 30% reduction in surface tension at droplet activation would produce a 20% increase in the cloud droplet number that could in turn lead to a change in cloud radiative forcing of up to –1 W m^–2^. Prisle *et al.*^16^ implemented different parameterisations of surfactant effects into a global circulation model and found that the resulting effects on the cloud droplet number could produce changes in cloud short-wave radiative forcing of –0.3–3%.

Predictions from the monolayer model are overall more in line with the results of Ruehl *et al.*^9^ and Ovadnevaite *et al.*,^10^ compared to Gibbs surface thermodynamics. In the latter case, this is not entirely unexpected, since the monolayer model discussed here shares some phenomenological features with that presented by Ovadnevaite *et al.*,^10^ although overall the monolayer model has a simpler construction and relies on fewer specific assumptions.^27^ Both frameworks assume a surface layer with finite thickness. In the monolayer model, the surface thickness is predicted from solution mixing properties, while Ovadnevaite *et al.*^10^ used an assumption based on organic C–C bond lengths, similar to that used by Prisle *et al.*^70^ and Walz *et al.*^80^,^81^ Both models evaluate the aqueous droplet surface tension as an average of individual compound surface tensions weighted according to volume fractions. The monolayer model furthermore accounts for the effect of dissociated salts on surface tension. A major difference between the two frameworks is that molecules in the surface layer are considered by Ovadnevaite *et al.*^10^ to form patches of two fully separated phases, while a single mixed phase is assumed in the monolayer model framework.

On the other hand, the monolayer model is more versatile than the approach presented by Prisle *et al.*^6^ or the compressed film model of Ruehl *et al.*,^9^ which both assume complete phase separation of organic- and water-rich phases. Ruehl *et al.*^9^ report in their Table S2† an average droplet wet diameter at activation of 1.8 ± 0.15 μm for 150 nm dry particles consisting of a 50 nm ammonium sulphate core with a succinic acid shell. Assuming densities of 1.56 and 1.77 g cm^–3^ for SCA and AS, respectively, these dry particles have an SCA mass fraction of 0.96. From Fig. S4† of Ruehl *et al.*,^9^ droplets at activation have surface tensions modeled from their Szyszkowski and compressed film models of 64.7 and 71.2 mN m^–1^, respectively. For a 150 nm SCA–NaCl dry particle with a SCA mass fraction of 0.96, the Gibbs and monolayer models of this work predict critical droplet surface tensions of 71.6 and 71.2 mN m^–1^, respectively. Therefore – keeping in mind that the salts are different between the particle mixtures in each work – the small change in critical droplet surface tension for SCA–NaCl particles in this work is overall consistent with the results of Ruehl *et al.*^9^

When considering various recently proposed approaches to model surface–bulk partitioning and CCN activation,^27^ the monolayer model provides a viable and physically transparent alternative to both the more simplified and more complex approaches, including Gibbs models.

## 4 Conclusions

The monolayer droplet model of Malila and Prisle^27^ was successfully used to predict the bulk/surface partitioning, surface composition, and surface thickness of aqueous droplets comprising several surface active organic aerosol model systems. Combined with equilibrium Köhler theory, the CCN activity of these surface active model aerosol systems was calculated for a range of dry particle sizes and surface active organic mass fractions, using both the monolayer model and a traditional Gibbs surface framework.

Underpinning the CCN activity of surface active aerosols in the two models is an interplay of several mechanisms, including species and droplet mixing state-dependent surface partitioning, surface tension reduction, dilution, and changing surface/bulk volume ratios of aqueous droplets as they grow and are activated. This makes it complicated to unequivocally establish conditions for which surfactant effects on cloud droplet activation thermodynamics are significant or not.

The monolayer model predicts CCN activity comparable to that of the Gibbs model despite having a conceptually different representation of droplet surface thermodynamics and requiring fewer component- and composition-specific inputs. Overall, droplets are predicted to be activated at lower critical supersaturations, meaning the surface active aerosols are predicted to be more CCN active with the monolayer model. Due to the physical limitations on surface partitioning imposed by the finite surface volume and component densities in the monolayer model, droplet bulk concentrations at activation are predicted to be higher and may even exceed the critical micelle concentration, something which is typically not seen for predictions from the Gibbs models. A comprehensive evaluation of micelle effects in activating droplets will however require a fully thermodynamically consistent extension of the current framework.

The CCN activity predicted by the two droplet models was compared to measurement data where possible. The measured CCN activity of particles comprising the stronger surfactants SDS and NAFA more closely matched the Gibbs model results, whereas measurements for poplar and ragweed pollenkitt fell in between the predictions of the two models. For SCA particles, the models each match one set of measurements in the literature.

The overall good performance of the monolayer model for complex NAFA and pollenkitt particles demonstrates one of the major advantages of the monolayer model over the Gibbs models. Being self-contained and requiring no specific mixing properties in terms of aqueous activity coefficients, the monolayer model is much more readily applicable for complex atmospherically relevant systems where compound and composition-specific data are not available.

## Conflicts of interest

There are no conflicts to declare.

## Supplementary Material

Supplementary information
