# Supplementary material for: Cloud droplet activation of organic–salt mixtures predicted from two model treatments of the droplet surface
Source: Environ Sci Process Impacts. 2018 Oct 10;20(11):1611–29. doi: 10.1039/c8em00345a (PMC6716451; doi:10.1039/c8em00345a)
Supplement: Supplementary file 1 [file EM-020-C8EM00345A-s001.pdf]

Electronic Supplementary Material (ESI) for Environmental Science: Processes & Impacts.
This journal is © The Royal Society of Chemistry 2018

Electronic Supplementary Information for "Cloud droplet activation of organic–salt mixtures predicted from two model treatments of the droplet surface"

Jack J. Lin, Jussi Malila, and Nønne L. Prisle

Nano and Molecular Systems Research Unit
P. O. Box 3000, FI-90014 University of Oulu

September 20, 2018

S1 Model sensitivity

As obvious from eqn. (1), the monolayer model is sensitive to the parametrisations used for the mixture as well as pure compound surface tensions. To some extent, the same can be said about pure component molecular volumes and densities of liquid mixtures, although due to the pseudobinary assumption applied in this work, there are no nonlinear dependencies which could explain features such as the non-monotonous dependence of the NAFA surface fraction in Fig. 10. To illustrate model sensitivity, we consider here two cases: i) dependence of results for NAFA–NaCl system on the surface tension parametrisation, and ii) dependence of the model predictions on the value of surface tension at CMC, which is used to approximate pure liquid surface tension for SDS, when σ_{CMC} is decreased to 31 mN m^{-1}, a value that could be a more proper choice for pure SDS (see the supplement of Malila and Prisle[1]). For the former case, instead of the second-order mass fraction dependence of Szyszkowski parameters a and b given in Table 1, a simpler linear approximation

$$a/(\mathrm{mN\,m^{-1}}) = 10.46 - 4.810\epsilon_{\mathrm{NAFA}} \quad \text{and}$$
$$b/(\mathrm{kg\,m^{-3}}) = 0.5947 - 0.3278\epsilon_{\mathrm{NAFA}},$$

where ϵ_{NAFA} is the mass fraction of NAFA in dry particle, is used. Similar sensitivity analysis for the Gibbs model has recently been given elsewhere.[2] In both cases, we consider dry particle size of 150 nm.

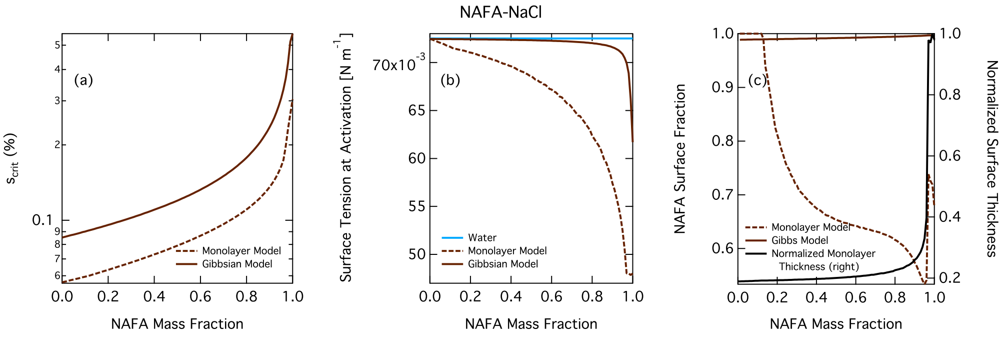

Figure S1: (a) Critical supersaturation, (b) surface tension at activation, and (c) NAFA surface fraction and normalized surface thickness calculated with alternative surface tension parameters for 150 nm NAFA particles as a function of NAFA mass fraction.

As can be seen from Figs. 8, 9, 10 and S1, at least for the 150-nm dry size, the form of surface tension parametrisation used strongly affects the shape of the NAFA surface fraction vs. NAFA mass fraction curve, but does not induce any significant changes. This further confirms the conclusions presented in the main text and shows that although details of predicted surface/bulk partitioning are sensitive to the assumed form of the surface tension function, predicted properties relevant for CCN activation are less so, as long as the surface tension parametrisation has a sensible form.

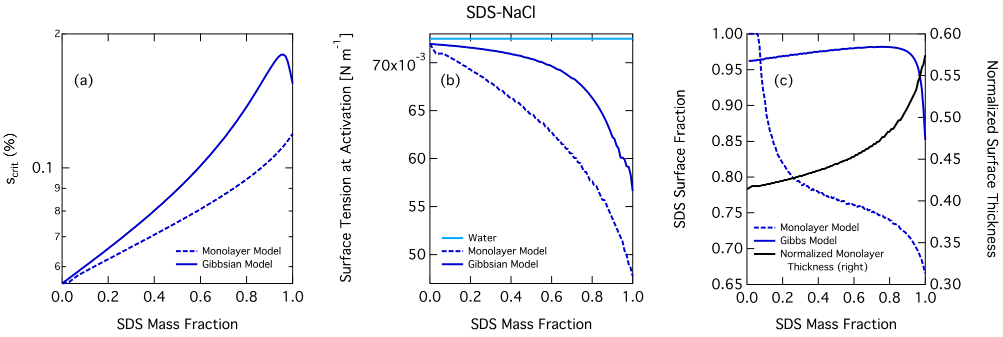

Figure S2: (a) Critical supersaturation, (b) surface tension at activation, and (c) SDS surface fraction and normalized surface thickness calculated with different different σ_{CMC} for 150 nm SDS particles as a function of SDS mass fraction.

In Fig. S2, model results for the SDS–NaCl mixture with reduced surface tension at CMC are given for a 150-nm dry particle. Comparison with Figs. 5, 6, and 7 shows only slight quantitative changes when ϵ_{SDS} is small. For higher SDS mass fractions in dry particle, though, differences are even qualitative, as concentration in droplet bulk does not reach CMC. This behaviour is, however, expected on basis of eqn. (1), as $x^s_{\mathrm{SDS}} < 1$ results in the modelled droplet surface tension. It can be concluded that the predictions of the monolayer model even for the activating droplets are sensitive to the assumed values of CMC and σ_{CMC} at high surfactant mass fractions.

S2 Growth factor at activation

Growth factor plots corresponding to Fig. 3 in the main text are shown here for SDS–NaCl, NAFA–NaCl, ragweed pollenkitt–$(NH_4)_2SO_4$, and poplar pollenkitt–$(NH_4)_2SO_4$.

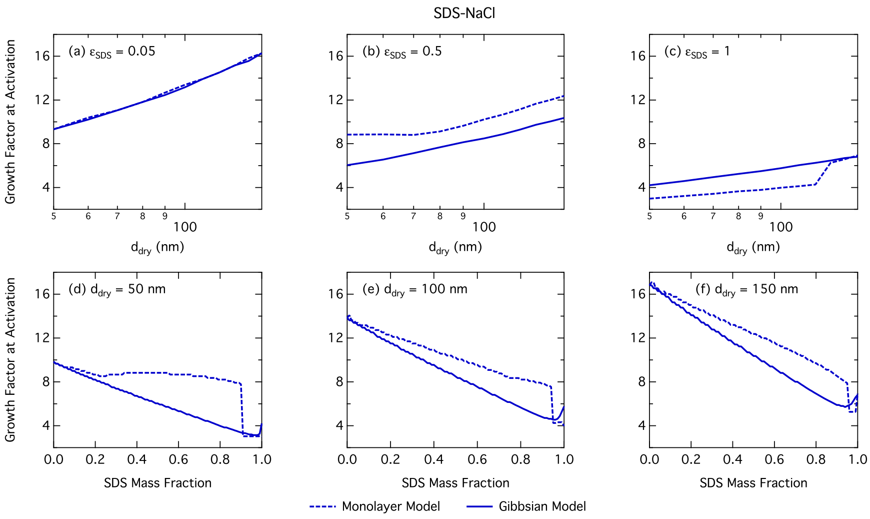

Figure S3: Growth factor at activation calculated by the monolayer and Gibbsian model as a function of dry particle size for SDS mass fractions (a) 0.05; (b) 0.5; (c) 1 and as a function of SDS mass fraction for dry particle sizes (d) 50 nm; (e) 100 nm; and (f) 150 nm.

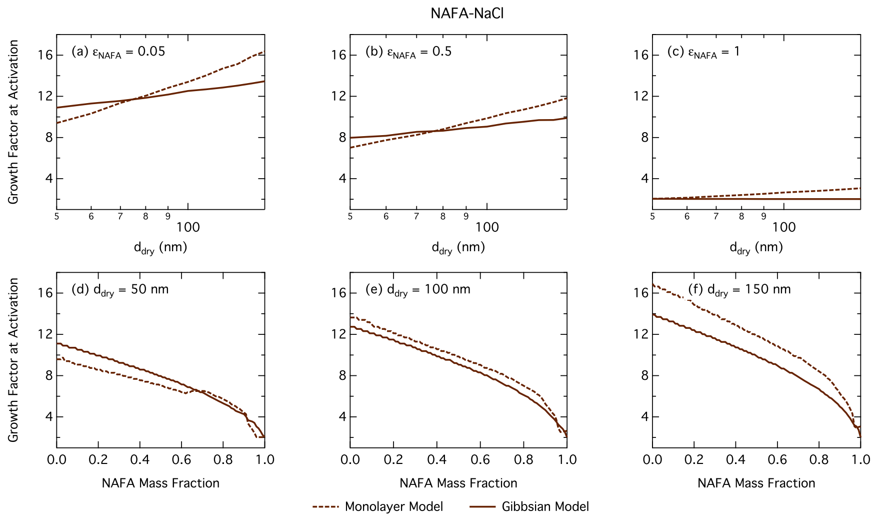

Figure S4: Growth factor at activation calculated by the monolayer and Gibbsian model as a function of dry particle size for NAFA mass fractions (a) 0.05; (b) 0.5; (c) 1 and as a function of NAFA mass fraction for dry particle sizes (d) 50 nm; (e) 100 nm; and (f) 150 nm.

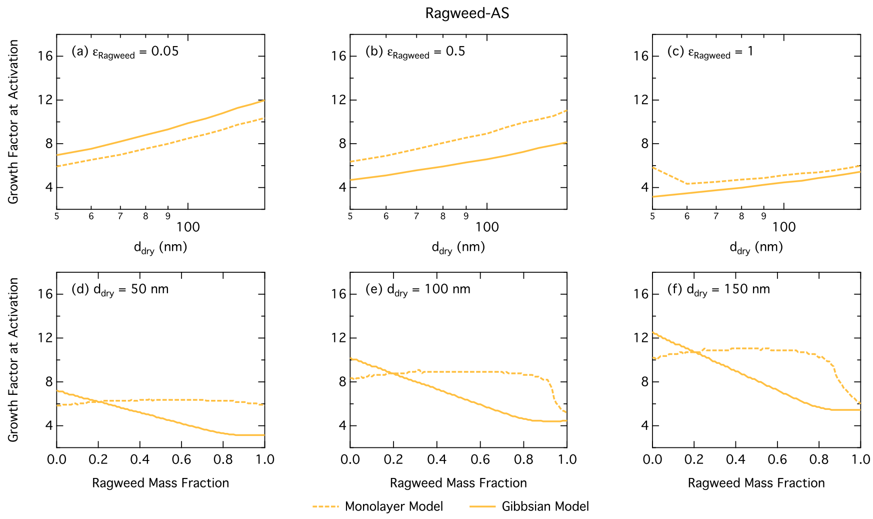

Figure S5: Growth factor at activation calculated by the monolayer and Gibbsian model as a function of dry particle size for ragweed pollenkitt mass fractions (a) 0.05; (b) 0.5; (c) 1 and as a function of pollenkitt mass fraction for dry particle sizes (d) 50 nm; (e) 100 nm; and (f) 150 nm.

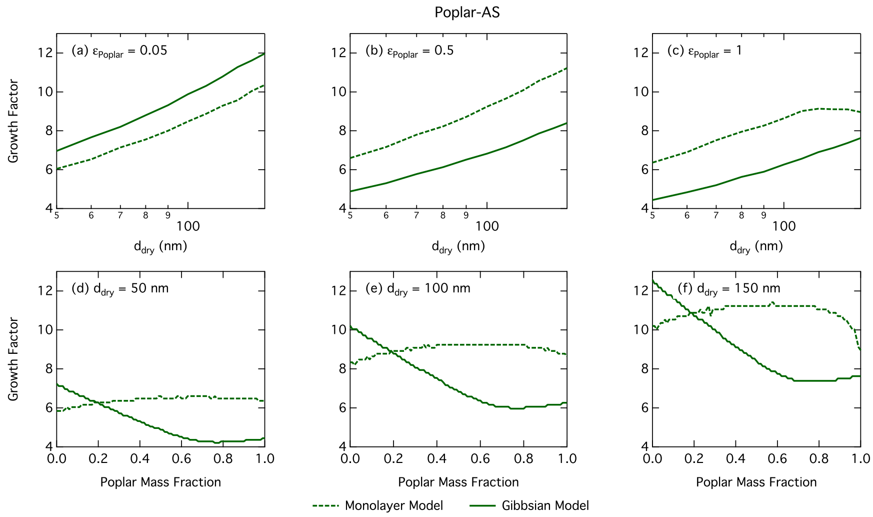

Figure S6: Growth factor at activation calculated by the monolayer and Gibbsian model as a function of dry particle size for poplar pollenkitt mass fractions (a) 0.05; (b) 0.5; (c) 1 and as a function of pollenkitt mass fraction for dry particle sizes (d) 50 nm; (e) 100 nm; and (f) 150 nm.

S3 Poplar pollenkitt

In this section, the figures for poplar pollenkitt analogous to those for ragweed pollenkitt are shown. We refer the reader to the discussion in section 3.2.2 in the main text.

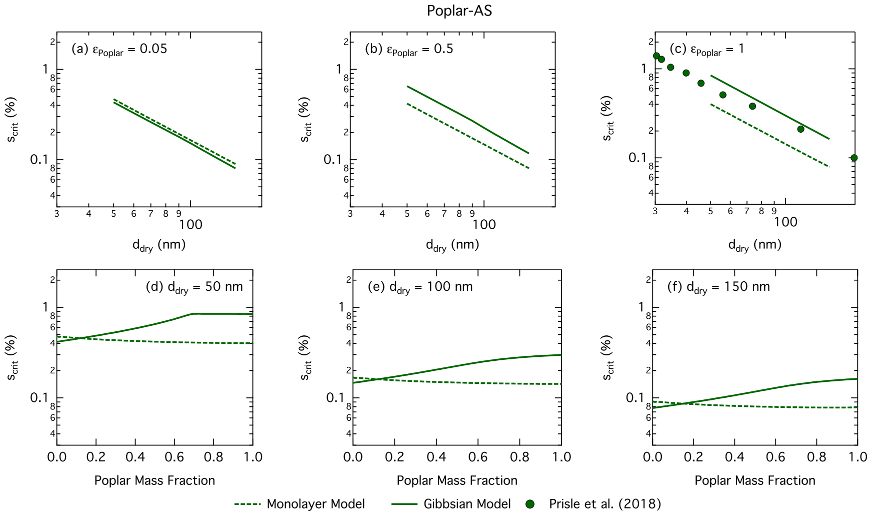

Figure S7: Critical supersaturations calculated by the monolayer and Gibbsian model as a function of dry particle size for poplar pollenkitt mass fractions (a) 0.05; (b) 0.5; (c) 1 and as a function of pollenkitt mass fraction for dry particle sizes (d) 50 nm; (e) 100 nm; and (f) 150 nm. Measured critical supersaturations as a function of pure poplar pollenkitt dry particle size from Prisle *et al.* [3] are also shown in panel (c).

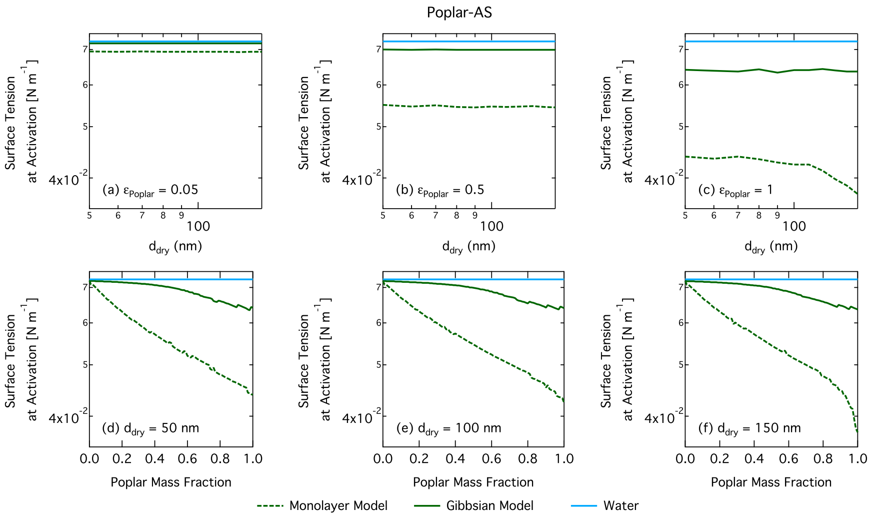

Figure S8: Surface tension at activation calculated by the monolayer and Gibbsian model as a function of dry particle size for poplar pollenkitt mass fractions (a) 0.05; (b) 0.5; (c) 1 and as a function of pollenkitt mass fraction for dry particle sizes (d) 50 nm; (e) 100 nm; and (f) 150 nm.

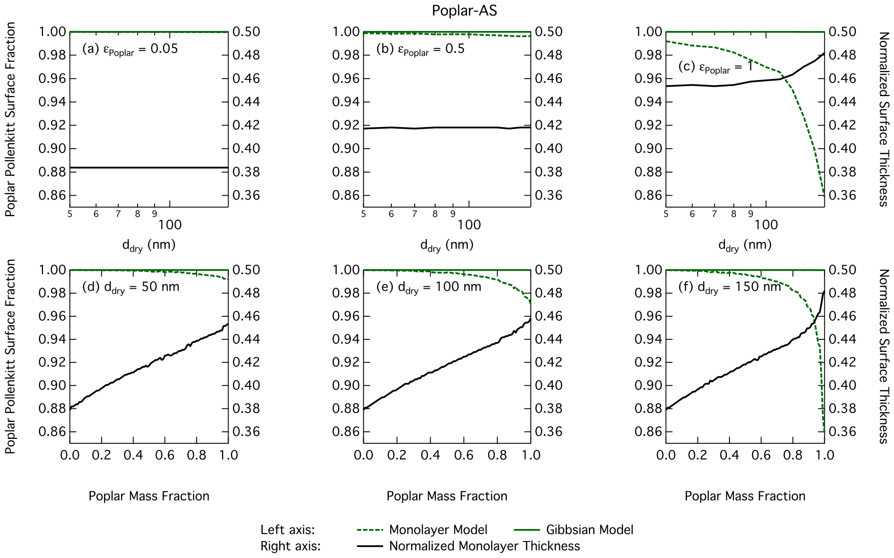

Figure S9: Droplet poplar pollenkitt surface fraction on the left axes calculated by the monolayer and Gibbsian model and surface thickness from the monolayer model normalized to the thickness of one poplar pollenkitt monolayer on the right axes as a function of dry particle size for poplar pollenkitt mass fractions (a) 0.05; (b) 0.5; (c) 1 and as a function of pollenkitt mass fraction for dry particle sizes (d) 50 nm; (e) 100 nm; and (f) 150 nm.

References

1 J. Malila and N. L. Prisle, *Journal of Advances in Modeling Earth System*, submitted.

2 N. L. Prisle and B. Molgaard, *Atmospheric Chemistry and Physics Discussions*, 2018, **2018**, 1–23.

3 N. L. Prisle, J. J. Lin, S. K. Purdue, H. Lin, J. C. Meredith and A. Nenes, *Atmospheric Chemistry and Physics Discussions*, 2018, **2018**, 1–26.
